# N-Acetyl-Heparin Attenuates Acute Lung Injury Caused by Acid Aspiration Mainly by Antagonizing Histones in Mice

**DOI:** 10.1371/journal.pone.0097074

**Published:** 2014-05-09

**Authors:** Yanlin Zhang, Zanmei Zhao, Li Guan, Lijun Mao, Shuqiang Li, Xiaoxu Guan, Ming Chen, Lixia Guo, Lihua Ding, Cuicui Cong, Tao Wen, Jinyuan Zhao

**Affiliations:** 1 Research Center of Occupational Medicine, Third Hospital of Peking University, Beijing, China; 2 Beijing Institute of Hepatology, Beijing You'an Hospital affiliated with Capital Medical University, Beijing, China; The Hospital for Sick Children and The University of Toronto, Canada

## Abstract

Acute lung injury (ALI) is the leading cause of death in intensive care units. Extracellular histones have recently been recognized to be pivotal inflammatory mediators. Heparin and its derivatives can bind histones through electrostatic interaction. The purpose of this study was to investigate 1) the role of extracellular histones in the pathogenesis of ALI caused by acid aspiration and 2) whether N-acetyl-heparin (NAH) provides more protection than heparin against histones at the high dose. ALI was induced in mice via intratracheal instillation of hydrochloric acid (HCl). Lethality rate, blood gas, myeloperoxidase (MPO) activity, lung edema and pathological changes were used to evaluate the degree of ALI. Heparin/NAH was administered intraperitoneally, twice a day, for 3 days or until death. Acid aspiration caused an obvious increase in extracellular histones. A significant correlation existed between the concentration of HCl aspirated and the circulating histones. Heparin/NAH (10 mg/kg) improved the lethality rate, blood gas, MPO activity, lung edema and pathological score. At a dose of 20 mg/kg, NAH still provided protection, however heparin tended to aggravate the injury due to hemorrhagic complications. The specific interaction between heparin and histones was verified by the binding assay. In summary, high levels of extracellular histones can be pathogenic in ALI caused by acid aspiration. By neutralizing extracellular histones, heparin/NAH can offer similar protection at the moderate doses. At the high dose, NAH provides better protection than heparin.

## Introduction

Acute lung injury (ALI) is characterized by refractory hypoxemia, increased alveolar–capillary permeability and uncontrolled overwhelming inflammatory responses. Acute respiratory distress syndrome (ARDS) is the final stage of ALI with a mortality rate of ∼40% or more, and is the leading cause of death in intensive care units [1], [2]. Furthermore, the severity of ALI is strongly associated with the incidence of multiple organ failure (MOF) [3], [4]. Aspiration pneumonia is a main risk factor for ALI/ARDS. A recent prospective cohort study showed that more than 10% of ALI/ARDS cases are associated with a witnessed aspiration [5]–[7].

The high mortality associated with ALI/ARDS indicates that the key mechanism of pathogenesis is unclear. With the exception of protective ventilation strategies for the lungs, interventions for ALI/ARDS are less purposeful [8], [9]. Rationally, a better understanding of the pathogenic mechanism of ALI/ARDS may help in the development of potentially effective therapies.

Extracellular histones have recently been recognized as pivotal mediators of lethal systemic inflammatory diseases, both infectious and noninfectious, including sepsis, acute ischemia-reperfusion injury and trauma [10]–[12]. Increased levels of extracellular histones and nucleosomes have been observed during acute inflammatory states. Xu et al identified extracellular histones as major mediators of death in sepsis. Histones are markedly released in septic models of mice, which can then trigger uncontrolled inflammation leading to death [13]. Abrams et al demonstrated that large quantities of nucleosomes are released into the circulation in patients with severe trauma and these nucleosomes are further degraded into histones. The circulating histones can mediate distant organ damage and even induce ALI and MOF [14].

Heparin, a highly sulfated proteoglycan, has long been used as a potent blood anticoagulant. Additionally, heparin and its derivatives have been shown to possess anti-inflammatory effects and protective properties [15], [16], which may result from their ability to bind histones through electrostatic interactions of high affinity [17]. Unfortunately, only moderate doses of heparin can attenuate injuries, high doses of heparin can be harmful due to complication of disseminated hemorrhage [18]–[20]. It is reasonable to suggest that chemically modified heparin derivatives, devoid of anticoagulant activity, may be more useful than heparin for controlling inflammation caused by histones.

The purpose of this study was to investigate 1) the role of extracellular histones in the pathogenesis of ALI caused by acid aspiration and 2) whether a high dose of N-acetyl-heparin (NAH) provides more protection against histones during ALI than does a high dose of heparin.

## Materials and Methods

### Reagents

Goat antibodies to histone H4 were purchased from Cell Signaling Technology (MA, USA). Calf thymus histones, heparin, NAH and heparin agarose were purchased from Sigma (Dorset, UK). The myeloperoxidase (MPO) detection kit was purchased from Jiancheng Biological Co. (Nanjing, China). A Cell Death Detection ELISA^PLUS^ was purchased from Roche Diagnostics (Indianapolis, USA) and used to measure circulating nucleosomes. The histone H4 detection kit was purchased from USCN Life Science Inc. (Wuhan, China). An activated partial thromboplastin time (APTT) detection kit was purchased from GenMed Scientifics Inc. (MA, USA).

### Preparation of Anti-histone H4 Antibody

Mouse anti-histone H4 Ab (anti-H4) was prepared following the previously described protocol [21]. Nonspecific mouse IgG was used as the control. The anti-H4 antibody (20 mg/ml) and the IgG control antibody (20 mg/ml) were injected via the tail vein once just prior to acid aspiration.

### Animals

Six to eight week-old male C57BL/6 mice, weighing 18–20 g, were purchased from the Experimental Animal Center of Peking University (Peking, China). Mice were housed in an air-conditioned room at 25°C with a 12 hours dark–light cycle. Upon arrival, mice were allowed to acclimate for 3 days before the experiment. All experimental protocols in this study were approved by the Institutional Animal Care and Use Committee of Health Sciences Center, Peking University (protocol no. LA201284). All procedures strictly followed institutional and federal guidelines. Efforts were made to minimize animal suffering, including administering anesthesia before surgery, administering medication for pain relief after surgery, and euthanasia by intravenous injection of ketamine and xylazine followed by cervical dislocation.

### Acid Aspiration Induced ALI

Before the induction of ALI, mice were fasted overnight but allowed water ad libitum. Mice were anesthetized by intraperitoneal (ip) injection of sodium pentobarbital (50 mg/kg) and placed supine at a 60° angle against choke. Lung injury was induced by intratracheal instillation of hydrochloric acid (HCl) (2 µl g^−1^) over 30 seconds, via the tracheostomy, at the level of the carina. HCl was diluted with 0.9% (w/v) normal saline. A concentration of 0.3 mol/l was used for the study of survival rate and 0.1 Mol/l was used for the study of injury degree. An equivalent amount of normal saline was administered intratracheally to control mice using same procedure as above [22]. After the operation, mice received subcutaneous injection of carprofen (3 mg/kg, twice a day, for 2 days) to relieve pain. Mice were allowed to recover on a heated pad and permitted access to food and water.

### Dose Response Analysis of Concentration of Acid Aspirated and Level of Circulating Nucleosomes or Histones

Lung injury was induced by intratracheal instillation of HCl (2 µl g^−1^) in mice. The concentrations of acid aspirated were 0.01 mol/l, 0.1 mol/l, 0.3 mol/l and 0.5 mol/l. The control mice underwent the same procedure with intratracheal instillation of equivalent normal saline. Whole blood was sampled in 11 mM sodium citrate, 6 and 12 hours after acid aspiration, and then centrifuged for plasma collection (1,000 r/min, 10 min). The circulating nucleosomes were measured with a Cell Death Detection ELISA^PLUS^ (Roche Diagnostics). Histone H4 was measured with an H4 detection kit (USCN life science Inc.) and Western blot.

### Survival Rate Analysis

The lethality rate was considered as a key mark for the severity of ALI. After the injury was induced by aspiration of lethal dose of acid (0.3 Mol/l, 2 µl g^−1^), mice were monitored every 4 hours for 96 hours. Lethargy, hunched gait, eye discharge and piloerection were recorded. When mice began to show signs of severe distress, such as labored breathing, severe eye discharge, piloerection, and non-responsiveness to touch, they were humanely euthanized by injecting ketamine (100 mg/kg) and xylazine (8 mg/kg) via the tail vein followed by cervical dislocation. The death was recorded as acid aspiration induced mortality.

### Treatment with Heparin and NAH

After intratracheal instillation of HCl (2 µl g^−1^), different doses of heparin/NAH (2.5, 5, 10, 20 mg/kg) were injected ip, twice a day, for 3 days or until death. The control and the untreated mice received ip injection of equivalent normal saline. After the mice were euthanized, the regional necropsies were performed by a pathologist to determine whether abnormal hemorrhagic foci were present, such as gastrointestinal bleeding, hemorrhage in abdominal cavity, hemothorax, or retroperitoneal hematoma.

### Blood Gas Analysis

After the experimental protocol was completed, mice were anesthetized by ip injection of sodium pentobarbital (50 mg/kg). Prior to euthanasia, an abdominal aortic catheter with sodium citrate was inserted for sampling blood. The blood sample (0.1 ml) was analyzed at 37°C using a Ciba Corning-170 blood gas analyzer (Ciba Corning, Canada).

### Mouse Plasma APTT Assay

After ip administration of heparin/NAH for 3 days, mice were anesthetized by ip injection of sodium pentobarbital (50 mg/kg). Whole blood was collected in 11 mM sodium citrate by abdominal aortic puncture and centrifuged for plasma collection immediately. Mouse plasma APTT was measured by turbidimetry with APTT detection kit (GenMed Scientifics Inc.).

### Western Blot of Mouse Plasma Histones

Whole blood was collected in 11 mM sodium citrate and plasma was isolated by centrifugation. Protein concentration was determined with the Bicinchoninic Acid Protein Assay Kit (Sigma, MO, USA). Equal amount of plasma proteins (100 µg) were mixed with loading buffer and subjected to electrophoresis using 12% (w/v) SDS-polyacrylamide gels. Separated proteins were transferred onto nitrocellulose membranes (Hybond-C Extra, Amersham). The membranes were then incubated with anti histone H4 antibody.

### MPO Measurement of Lung Tissue

After the experimental protocol was completed, lung samples were rapidly obtained from the left lower lobes for measurement of MPO. The samples were homogenized with 0.9% (w/v) normal saline and centrifuged. The supernatant was detected by an MPO detection kit at 460 nm wavelength with a 1601-UV–Visible Spectrophotometer (Shimadzu, Japan).

### Analysis of Lung Edema

Samples of mouse lung tissues were obtained from the right upper lobes. After the lung tissues were weighed (wet weight), the samples were dried in an oven at 60°C for 72 hours and weighed again (dry weight). The ratio of wet/dry lung weight was used to indicate the degree of lung edema.

### Lung Histopathology Analysis

Lung samples were obtained from the right lower lobes. The stained sections were scored by pathologists who were blinded to the experimental protocol. The degree of microscopic injury was scored based on the following variables: hemorrhage, interstitial edema, necrosis, neutrophil infiltration and atelectasis. The severity of injury was judged by previously reported criteria [23]. Three microscopic visual fields were selected randomly for each lung section.

### Binding Assay of Histones and Heparin

Whole blood was collected from mice 12 hours after acid aspiration (0.1 mol/l, 2 µlg^−1^) and was centrifuged to collect plasma. The plasma from the injury group was incubated with a heparin-agarose solution or an agarose solution at 37°C for 30 minutes. After incubation the plasma was separated from the heparin-agarose or agarose solution by centrifugation (3,000 r/min, 5 min) and measured with a histone H4 ELISA kit.

### Statistical Analysis

The results are presented as mean ±SD. Statistical significance of differences among groups was determined by ANOVA followed by the Student-Newman-Keuls test. The nonparametric Kruskal–Wallis test was used when comparing pathological scores. Animal survival time was analyzed with a log-rank test and correlation was analyzed with a Pearson test in Prism (GraphPad Software). A *p*-value <0.05 was regarded as significant.

## Results

### Dose response analysis of acid aspirated and the severity of ALI

The lung injury was induced by intratracheal instillation of HCl via the tracheostomy. In the preliminary experiment, different concentrations of HCl were used to induce ALI. The mice were observed every 4 hours for 72 hours after acid aspiration. The dose of 0.01 mol/l HCl (2 µl g^−1^) caused transient tachypnea and all mice (7/7) survived, while the dose of 0.1 mol/l (2 µl g^−1^) caused obvious dyspnea and 5 mice (5/7) survived for 72 hours. The dose of 0.3 mol/l (2 µl g^−1^) caused serious dyspnea and only 1 mouse (1/7) survived for 72 hours. The dose of 0.5 mol/l (2 µl g^−1^) caused very serious dyspnea and all mice (7/7) died within 24 hours (**Fig. S1**). Meanwhile liver (alanine transaminase, ALT; aspartate transaminase, AST) and renal (blood urea nitrogen, BUN; creatinine, Cre) function was not evidently impaired 12 hours after acid aspiration. The pathological changes in liver and kidney were also not obvious (**Fig. S2**). These data collectively indicate that lungs are much more susceptible to the toxicity of circulating histones than other organs, so respiratory failure may be the cause of death in early stage of ALI. Based on the data from the preliminary experiment, the dose of 0.3 mol/l (2 µl g^−1^) was chosen to study the lethality rate and 0.1 mol/l (2 µl g^−1^) was chosen to evaluate the degree of ALI by means of blood gas, MPO activity, lung edema and pathological changes.

### Change of nucleosomes and histones in circulation after acid aspiration

**Figure 1:**
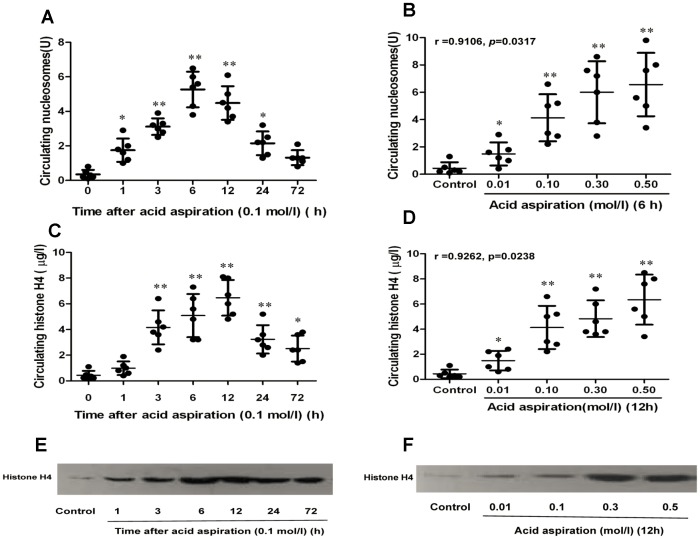
Change in circulating nucleosomes and histones during acid aspiration caused ALI in mice. The mice were challenged by HCl aspiration in different concentrations (0.01, 0.1, 0.3 and 0.5 mol/l, 2 µl g^−1^). Circulating nucleosomes were measured with ELISA kits at predetermined time points for dynamic change (1A) and dose response analysis (1B). Circulating histone H4 was measured by both ELISA and Western blot at predetermined time points for dynamic change (1C, 1E) and dose response analysis (1D, 1F). Data are presented as mean±SD (n = 6). The Western blot results are representative of three similar experiments. *p<0.05 vs. the control group, **p<0.01 vs. the control group.

Nucleosomes, which are composed of histones and DNA, were nearly undetectable in the normal mouse circulation. As shown in Figure 1, the levels of nucleosomes and histones were very low in the plasma from the control mice. One hour after acid aspiration, the increase of nucleosomes in circulation was evident, especially when the concentration of HCl exceeded 0.1 mol/l (2 µl g^−1^) (**Figure 1A**). Longitudinal analysis showed that the level of plasma nucleosomes reached a peak in 6 hours and then decreased quickly, degrading into histones and DNA. Similarly, extracellular histone H4 started to increase gradually 1 hour after acid aspiration (**Figure 1C**). Notably high levels of histones were detected at 3 and 6 hours, and reached a peak at 12 hours after aspiration and then decreased gradually. Even after 72 hours, the level of histones in HCl-treated mice was still much higher than in control mice.

As shown in the preliminary experiment, the concentration of HCl aspirated had a direct effect on the degree of lung injury and on the lethality rate. Simultaneously the concentration of HCl aspirated also determined the amount of circulating histones and nucleosomes. As shown in **Figure 1B and 1D**, the Pearson correlation analysis revealed a significant correlation between the concentrations of HCl aspirated (ranging from 0.01 to 0.5 mol/l) and circulating nucleosomes (r = 0.9106, *p* = 0.0317), and circulating histone H4 (r = 0.9262, *p* = 0.0238).

The difference in circulating histones was also validated by Western blot analysis (**Figure 1E, 1F**).

### Effect of anti-H4 antibody on the survival rates in mice

**Figure 2:**
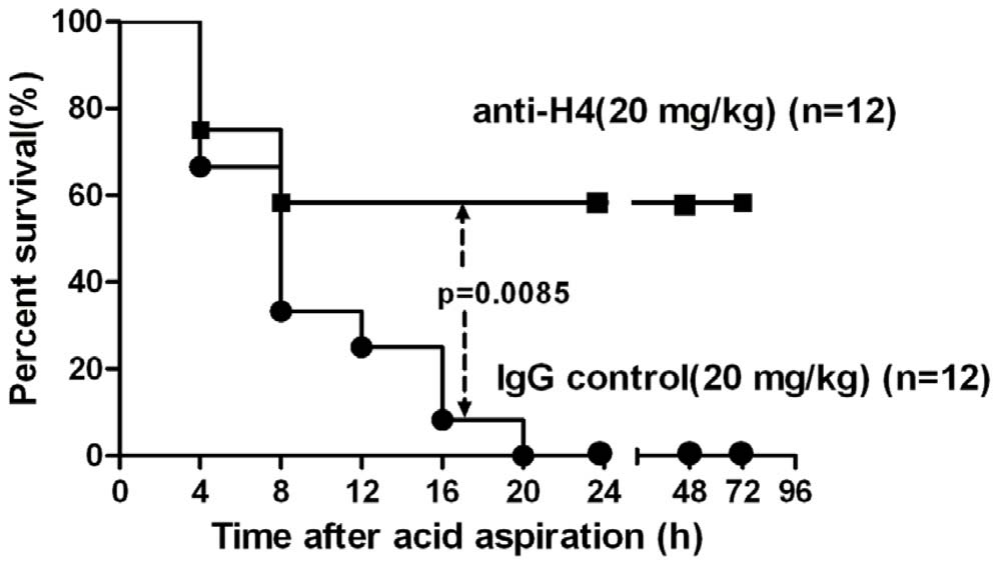
Effect of anti-H4 antibody on survival rates in mice with acid aspiration caused ALI. Anti-H4 antibody (20 mg/ml) or the IgG control (20 mg/ml) were given once intravenously just before acid aspiration (0.3 mol/l, 2 µl g^−1^). The untreated mice all died within 24 hours (n = 12). Eight of 12 mice Pre-treated with anti-H4 antibody survived the challenge for 72 hours, but the IgG control did not improve the survival rates. Log-rank test was used for comparison of survival time. *P*<0.05 is viewed as statistically significant.

To verify whether the extracellular histones are the major mediators of damage, mice were pre-treated with specific anti-H4 antibody or nonspecific mouse IgG just prior to acid aspiration. As shown in **Figure 2**, all mice died within 24 hours (n = 12) after aspiration of a lethal dose of acid (0.3 mol/l, 2 µl g^−1^). However, 8 of 12 mice that were pre-treated with intravenous anti-H4 (20 mg/kg) survived for 72 hours, whereas pre-treatment with the non-specific mouse IgG (20 mg/kg) did not improve the survival rates. The difference of survival rates between the mice treated with anti-H4 and those treated with IgG was statistically significant by the log-rank test (*p* = 0.0085).

### Effect of heparin/NAH on the survival rates in mice

**Figure 3:**
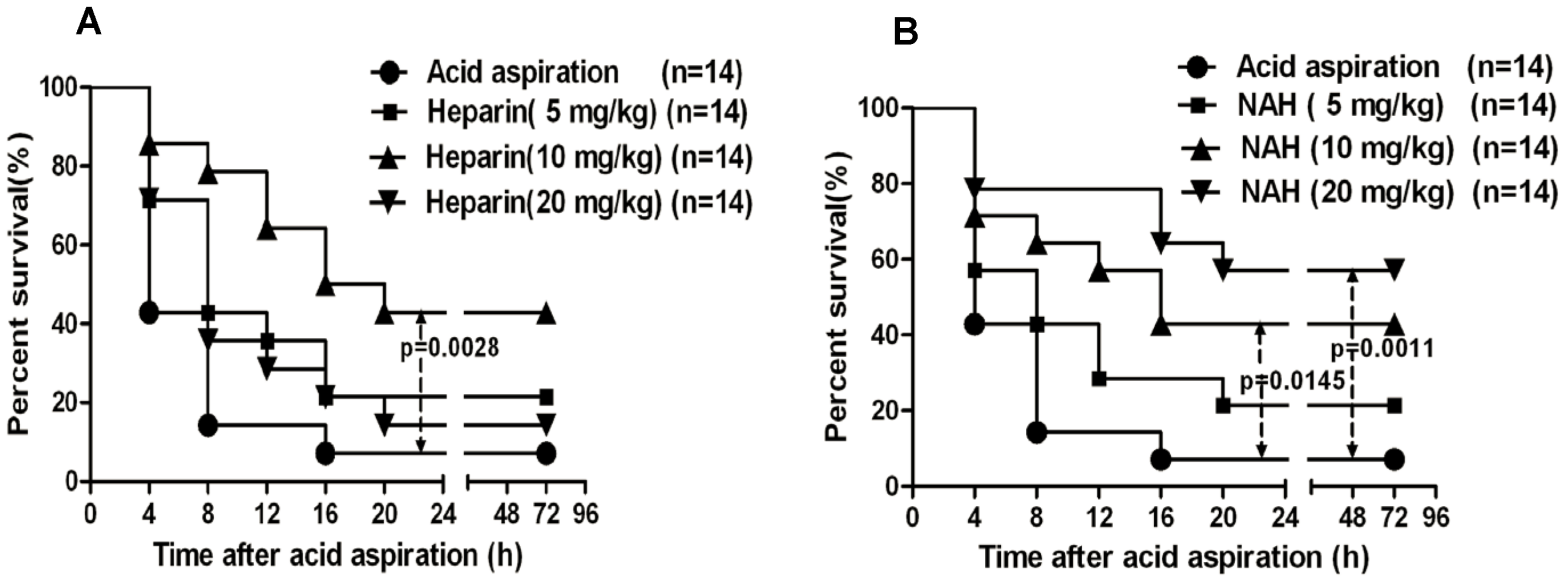
Effect of heparin/NAH on survival rates in mice with acid aspiration caused ALI. After mice were challenged by lethal acid aspiration (0.3 mol/l, 2 µlg^−1^), either heparin or NAH was injected ip simultaneously, twice a day, for 3 days or until death. The control and the untreated mice underwent the same procedure and were injected with equivalent normal saline. The survival rates were recorded every 4 hours for 72 hours. Heparin improved the survival rates (n = 14) (3A). NAH also relieved the lethality rates, especially at the high dose (n = 14) (3B). Log-rank test was used for comparison of survival time. *P*<0.05 is viewed as statistically significant.

After intratracheal instillation of HCl (0.3 mol/l, 2 µl g^−1^), mice were ip injected with heparin/NAH, twice a day, for 3 days or until death, in order to examine the protective effect of heparin/NAH on acid aspiration-induced lethality. Animal survival was recorded for 72 hours after acid aspiration. As shown in **Figure 3A**, the survival rates at 4 and 72 hours after acid aspiration were 42.9% and 7.1%, respectively, in mice treated by equivalent normal saline. Heparin could improve the survival rates. The dose of 5 mg/kg heparin slightly improved the survival rates at 4 and 72 hours, while 10 mg/kg heparin dramatically improved the survival rates to 85.7% and 42.9%, respectively. However when heparin dose reached 20 mg/kg, the survival rates decreased to 71.4% and 14.3%, respectively. As shown in **Figure 3B**, after acid aspiration treatment with 5 mg/kg NAH slightly improved the survival rates, while 10 mg/kg NAH significantly improved the survival rates at 4 and 72 hours to 71.4% and 42.9%, respectively. Unlike heparin, treatment with 20 mg/kg NAH further improved the survival rates to 78.6% and 57.1%, respectively.

### Effect of heparin/NAH on the degree of ALI in mice

**Figure 4:**
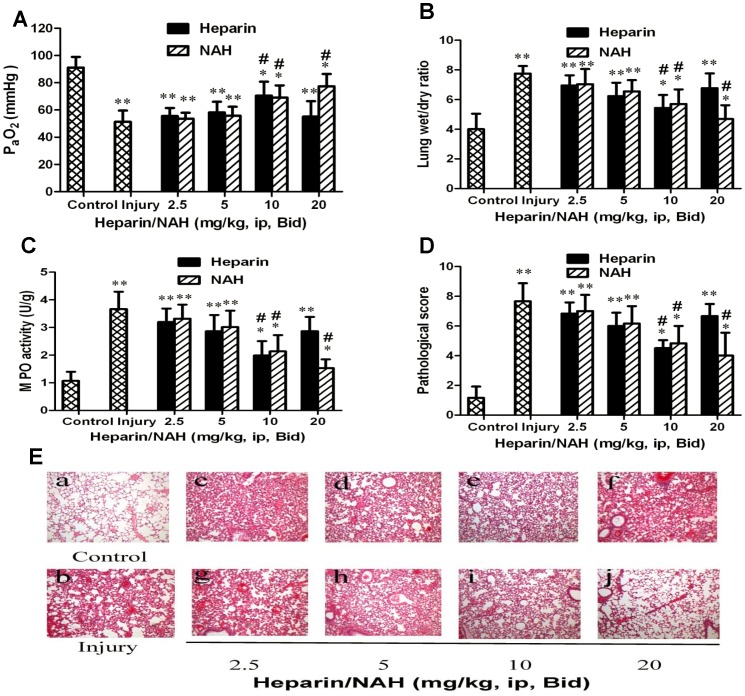
Effect of heparin/NAH on degree of acid aspiration caused ALI in mice. After acid aspiration (0.1 mol/l, 2 µl g^−1^), different doses of heparin/NAH (2.5, 5, 10, 20 mg/kg, ip) were injected, twice a day, for 3 days. Heparin/NAH improved blood gas (4A), lung edema (4B), MPO activity of lung tissue (4C) and pathological changes (4D, 4E). In Figure 4E: (a) control group, (b) acid aspiration group, (c, d, e, f) groups treated by heparin, (g, h, i, j) groups treated by NAH. Data are presented as mean±SD (n = 6). The hematoxylin and eosin (HE) stained lung sections are representative of 3 similar samples. Original magnification×200. *p<0.05 vs. the control group, **p<0.01 vs. the control group; **^#^** *p*<0.05 vs. the injury group, **^##^** *p*<0.01 vs. the injury group.

Mice were ip injected with heparin/NAH, twice a day, for 3 days after acid aspiration (0.1 mol/l, 2 µl g^−1^). The degree of ALI was evaluated by blood gas, MPO activity, lung edema and pathological changes. As shown in **Figure 4A**, hypoxemia was much more evident in the injury group than in the control group. Heparin/NAH improved the blood gas. The dose of 5 mg/kg slightly relieved the decrease in PaO_2_, while 10 mg/kg dramatically improved PaO_2_. At 20 mg/kg, NAH provided better protection than 10 mg/kg, whereas the protection provided by heparin nearly disappeared. The change in PaCO_2_ and pH was not evident among different groups at predetermined time points (data not shown).

Pulmonary edema is viewed as a hallmark of ALI. As shown in **Figure 4B**, acid aspiration caused evident lung edema, which was characterized by a high ratio of wet/dry lung weight. The dose of 5 mg/kg heparin/NAH slightly relieved the lung edema, while 10 mg/kg dramatically decreased the wet/dry lung weight ratio. At 20 mg/kg, NAH further improved the lung edema, however heparin did not improve the edema nearly.

Neutrophil activation is considered to be a major mechanism for the inflammation leading to lung injury. MPO is mainly released from neutrophils, which can promote killing the harmful bacteria. However, excess MPO can also damage host cells. As shown in **Figure 4C**, heparin/NAH could attenuate the lung inflammation. The dose of 5 mg/kg slightly relieved the MPO activity while 10 mg/kg had an obvious effect. When dose reached 20 mg/kg, NAH further decreased the lung MPO activity, but heparin mostly did not.

Acid aspiration caused evident pathological changes, such as widespread thickened alveolar interstitial, severe hemorrhage in the alveolus, alveolus collapse and obvious inflammatory cell infiltration. As shown in **Figure 4D and 4E**, heparin/NAH could improve the pathological changes. The dose of 5 mg/kg relieved the pathological changes slightly and 10 mg/kg dramatically attenuated pathological injuries. When dose reached 20 mg/kg, NAH still afforded protection, but the protection provided by heparin nearly disappeared.

### Effect of heparin/NAH on abnormal hemorrhage in mice

**Figure 5:**
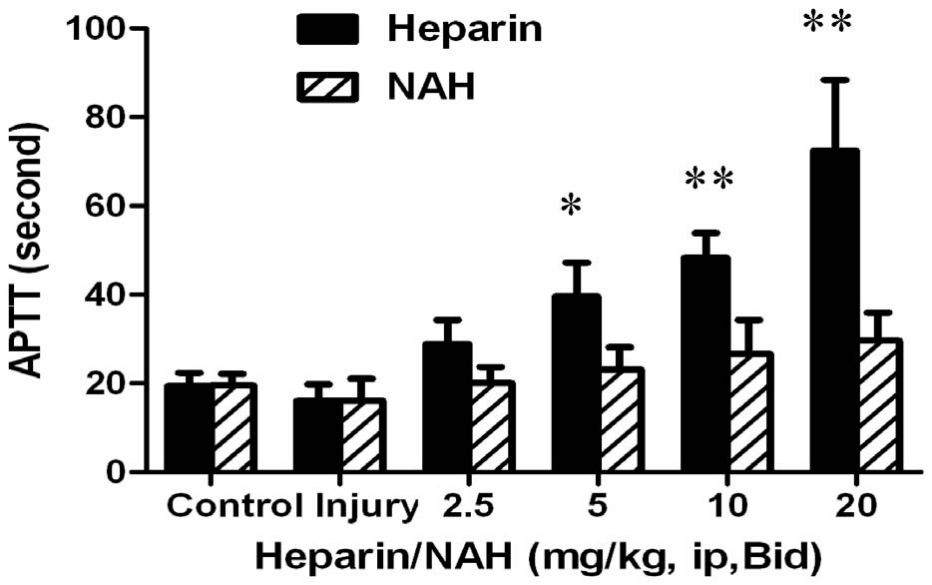
Effect of heparin/NAH on coagulation in mice with acid aspiration caused ALI. After acid aspiration (0.1 mol/l, 2 µl g^−1^), different doses of heparin/NAH (2.5, 5, 10, 20 mg/kg, ip) were injected, twice a day, for 3 days. When mice were anesthetized, whole blood was collected and centrifuged for plasma. Mice plasma APTT was measured by turbidimetry with an APTT detection kit. Data are presented as mean±SD (n = 6). *p<0.05 vs. the control group, **p<0.01 vs. the control group.

Heparin has been used as a potent anticoagulant, which can prolong APTT by binding to antithrombin III (AT III) and catalytically accelerating the inhibition of thrombin and factor Xa. APTT is a quantitative coagulation test of the intrinsic coagulation pathway. As shown in **Figure 5**, the mean baseline APTT value for the control mice was 19±2 seconds. Acid aspiration caused a mild shortening of the APTT, which might result from the partial coagulation activation. The prolongation of APTT was more and more evident as the heparin dose increased. The doses of 5 and 10 mg/kg prolonged the APTT to 40±6 and 48±5 seconds, respectively, about twice that of the control. The dose of 20 mg/kg dramatically prolonged the APTT to 73±12 seconds, about 3–4 times that of the control. Inevitably, the prolonged APTT increased the risk of bleeding. In contrast, the effect of NAH on APTT was much smaller compared with heparin. The APTT value was 25±3 seconds even when NAH dose reached 20 mg/kg.

After different doses of heparin/NAH (2.5, 5, 10, 20 mg/kg) were injected ip, twice a day, for 3 days or until death, regional necropsies were done to determine whether abnormal hemorrhagic foci were present. No abnormal hemorrhagic foci were found in the injury group, the 2.5 and 5 mg/kg heparin groups or the NAH groups. Gross view showed that 2 mice (2/14) had disseminated hemorrhage in abdominal cavity in 10 mg/kg heparin group while 5 mice (5/14) had disseminated hemorrhage in 20 mg/kg heparin group. The HE stained ileum sections showed that multifocal regions of hemorrhage were present within the muscular intestinal wall in the 10 and 20 mg/kg heparin groups (**Fig. S3**).

### Binding assay of histones and heparin

**Figure 6:**
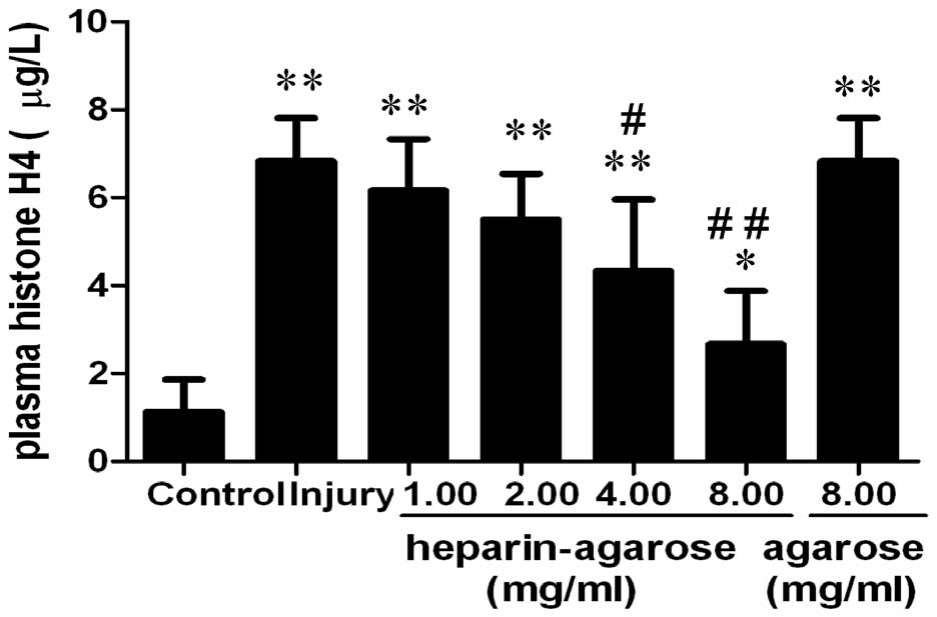
Binding assay of histones and heparin. Whole blood was collected and centrifuged for plasma 12(0.1 mol/l, 2 µl g^−1^). The plasma collected from the injury group was incubated with heparin-agarose solution or agarose solution at 37°C for 30 minutes. After incubation the plasma was separated from the heparin-agarose or agarose by centrifugation and measured with an H4 ELISA kit. *p<0.05 vs. the control group, **p<0.01 vs. the control group; **^#^** *p*<0.05 vs. the injury group, **^##^** *p*<0.01 vs. the injury group.

As shown above, heparin/NAH attenuated ALI caused by acid aspiration. To verify whether the protection of heparin/NAH was due to the histone-neutralizing effect, a binding assay of histones and heparin was done. As shown in **Figure 6**, the plasma extracellular histones increased sharply after acid aspiration. Treatment with a heparin-agarose solution could evidently decrease the level of plasma histones, but treatment with agarose only had no effect on plasma histones. This indicates that heparin can bind histones with high affinity and may block the interaction of histones and their ligands.

## Discussion

ALI is characterized by overwhelming inflammatory responses. Damage-associated molecular patterns (DAMPs) are considered to be another major pathway of uncontrolled inflammation in addition to the classic pathogen-associated molecular patterns (PAMPs), which include histones, mitochondrial DNA and formyl peptides [24]–[26].

In present study, the dose of 0.1 mol/l HCl (2 µl g^−1^) caused evident injury, while the dose of more than 0.3 mol/l (2 µl g^−1^) was lethal in mice. A significant correlation was found between circulating histones and the concentrations of HCl aspirated within the range of 0.01to 0.5 mol/l. However, blocking the circulating histones via neutralizing antibodies provided significant protection. Therefore it can be deduced that the circulating histones are the main mediators in acid aspiration caused ALI.

In innate immune responses, extracellular histones can form neutrophil extracellular traps (NETs) and have bactericidal activity [27], [28]. However, extracellular histones are a double-edged sword because they can also injure normal host tissues [13], [14]. In nucleosomes, the histone core interacts with the phosphodiester bonds of DNA, as shown by crystallography [29]. In blood circulation, phospholipids of pulmonary endothelial cells are the targets of circulating histones and have been shown to interact with histones [30]. Freeman et al showed that the circulating histones can accumulate on heparan sulphate in the capillary glycocalyx of the lungs [31]. The interaction of histones and endothelia can lead to increased permeability of cell membranes, followed by large inward ion currents and calcium overload, resulting in cell dysfunction and lung edema [32]–[34]. Additionally, NETs are not exclusively produced during severe infections; they have also been observed in transfusion-related acute lung injury (TRALI). NETs can injure the lung endothelia by extracellular histones, neutrophil granular proteins and a tangled web of extracellular DNA [35].

Similar to histones, heparin is also highly evolutionarily conserved. Histones can fight invading microbes through microbicidal properties while heparin can prevent the damage due to histones released from injured cells [36], [37].

The current study showed that moderate dose of heparin/NAH could not only improve the survival rates of mice challenged by lethal acid aspiration but also improve blood gas, MPO activity associated with neutrophil activation, lung edema and pathological changes. At the high dose of 20 mg/kg, NAH still provided protection, but heparin could be harmful. Hayashi et al demonstrated that after atrial fibrillation ablation, APTT is the main risk factor for bleeding complications in patients with continued anticoagulation therapy with Warfarin [38]. Warren et al reported a significant increase in the incidence of bleeding in patients with severe sepsis who were receiving high-doses of heparin and concomitant AT III [39]. As shown in the current study, 20 mg/kg heparin dramatically prolonged the APTT, while NAH only prolonged APTT slightly. The regional necropsies showed that the high dose of heparin caused abnormal hemorrhage such as disseminated hemorrhage in abdominal cavity and gastrointestinal bleeding. Thus it can be deduced that the high dose of heparin fails to provide protection in acid aspiration induced ALI mainly due to hemorrhagic complications.

Heparin and NAH are both highly negatively charged thus they can bind histones by electrostatic interaction. Freeman et al showed that heparan sulphate and heparin have similar effectiveness in preventing the binding of histones and pulmonary endothelia [31]. Moreover, chemically modified non-anticoagulant heparin derivates may exert greater affinity for histones since their affinity for AT III is reduced [40], [41]. The binding assay showed that heparin could bind histones through electrostatic interaction of high affinity, which might block the interaction of histones and endothelia. Therefore, the protection of heparin/NAH may be from the histone-neutralizing effect, especially at the high dose.

In addition to neutralizing extracellular histones, heparin/NAH may also antagonize inflammation by other mechanisms. Schmidt et al demonstrated that pulmonary endothelial glycocalyx degradation can contribute to the adhesion of neutrophils to the endothelial surface and inflammatory tissue injury. Glycocalyx degradation involves the specific loss of heparan sulfate and the activation of endothelial heparanase. Both heparin and NAH can prevent the degradation of glycocalyx since they are both the competitive antagonists of heparanase [42], [43].

In summary, high levels of circulating histones appear to be the major pathogenic mediator in ALI caused by acid aspiration. This suggests that extracellular histones may be potential therapeutic targets for ALI and possibly MOF. Both heparin and NAH can offer similar protection mainly through blocking of extracellular histones. However, when the dose exceeds a certain level, heparin can aggravate injury because of hemorrhagic complications, while NAH can still provide protection. Thus it is conceivable that non-anticoagulant heparin derivatives may offer novel treatment in ALI mediated by histones.

## Supporting Information

Figure S1 **Dose response analysis of the concentration of acid aspirated and the lethality rate, blood gas and pathological changes in lung.** Different concentrations of HCl were used to induce ALI (2 µl g^−1^, n = 7). After acid aspiration mice were monitored every 4 hours for 72 hours. The concentration of 0.01 mol/l caused transient polypnea and all mice (7/7) survived, while 0.1 mol/l caused obvious dyspnea and 5 mice (5/7) survived for 72 hours. The concentration of 0.3 mol/l caused serious dyspnea and only 1 mouse (1/7) survived for 72 hours. In the group of 0.5 mol/l all mice (7/7) died within 24 hours (S1A). After acid aspiration for 12 hours (n = 7), an abdominal aortic catheter was inserted for sampling blood. Hypoxemia was much more evident in the injury group than in the control group (S1B). *p<0.05 vs. the control group, **p<0.01 vs. the control group. After acid aspiration for 12 hours (n = 7), lung samples were obtained for histopathology analysis. The hematoxylin and eosin (HE) stained lung sections are representative of 3 similar samples(S1C). Original magnification×200.
(TIFF)

Figure S2 **Effect of the concentration of acid aspirated on the function and pathological changes of liver and kidney.** After acid aspiration for 12 hours or just before death (n = 7), blood, liver and kidney samples were obtained. Liver (aspartate transaminase, AST; alanine transaminase, ALT) and renal (blood urea nitrogen, BUN; creatinine, Cre) function was analyzed. Mean from control group was designated as 100% and the relative percentages were presented. A slight increase in BUN and AST was seen in the group of 0.5 mol/l (S2A). *p<0.05 vs. the control group. Unlike in the lung, pathological changes in liver and kidney were not obvious, and slight swelling could be seen in liver and kidney tubule cells in the groups of 0.3 and 0.5 mol/l (S2B, S2C). The HE stained sections are representative of 3 similar samples. Original magnification×200.
(TIFF)

Figure S3 **Effect of heparin on abnormal hemorrhage in mice.** After intratracheal instillation of HCl (0.3 mol/l, 2 µl g^−1^), heparin was injected ip, twice a day, for 3 days or until death. The regional necropsies showed that no abnormal hemorrhagic foci were found in the injury group, the 2.5 and 5 mg/kg heparin groups or the NAH groups. Gross view showed that 2 mice (2/14) had disseminated hemorrhage in abdominal cavity in 10 mg/kg heparin group while 5 mice (5/14) had disseminated hemorrhage in the 20 mg/kg heparin group (S3A). Meanwhile ileum samples were obtained for histopathology analysis. The multifocal regions of hemorrhage were present within the muscular intestinal wall in the 10 and 20 mg/kg heparin groups. The HE stained sections are representative of 3 similar samples (S3B). Original magnification×100.
(TIFF)
