# Supplementary figures and images for: N-Acetyl-Heparin Attenuates Acute Lung Injury Caused by Acid Aspiration Mainly by Antagonizing Histones in Mice
Source: PLoS One. 2014 May 9;9(5):e97074. doi: 10.1371/journal.pone.0097074 (PMC4016230; doi:10.1371/journal.pone.0097074)

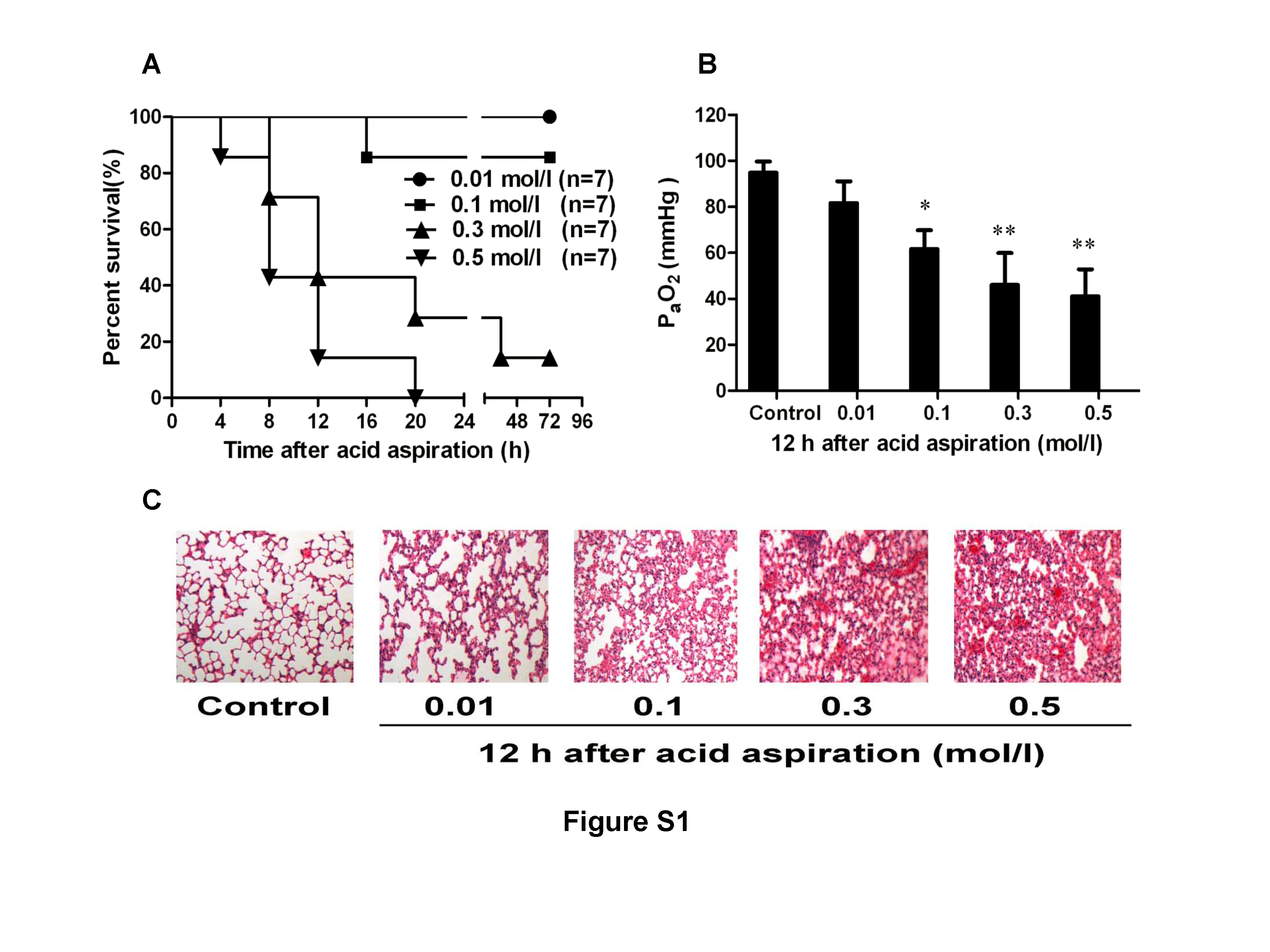

Supplement: Figure S1 — Dose response analysis of the concentration of acid aspirated and the lethality rate, blood gas and pathological changes in lung. Different concentrations of HCl were used to induce ALI (2 µl g−1, n = 7). After acid aspiration mice were monitored every 4 hours for 72 hours. The concentration of 0.01 mol/l caused transient polypnea and all mice (7/7) survived, while 0.1 mol/l caused obvious dyspnea and 5 mice (5/7) survived for 72 hours. The concentration of 0.3 mol/l caused serious dyspnea and only 1 mouse (1/7) survived for 72 hours. In the group of 0.5 mol/l all mice (7/7) died within 24 hours (S1A). After acid aspiration for 12 hours (n = 7), an abdominal aortic catheter was inserted for sampling blood. Hypoxemia was much more evident in the injury group than in the control group (S1B). *p<0.05 vs. the control group, **p<0.01 vs. the control group. After acid aspiration for 12 hours (n = 7), lung samples were obtained for histopathology analysis. The hematoxylin and eosin (HE) stained lung sections are representative of 3 similar samples(S1C). Original magnification×200. (TIFF) [file pone.0097074.s001.tiff]

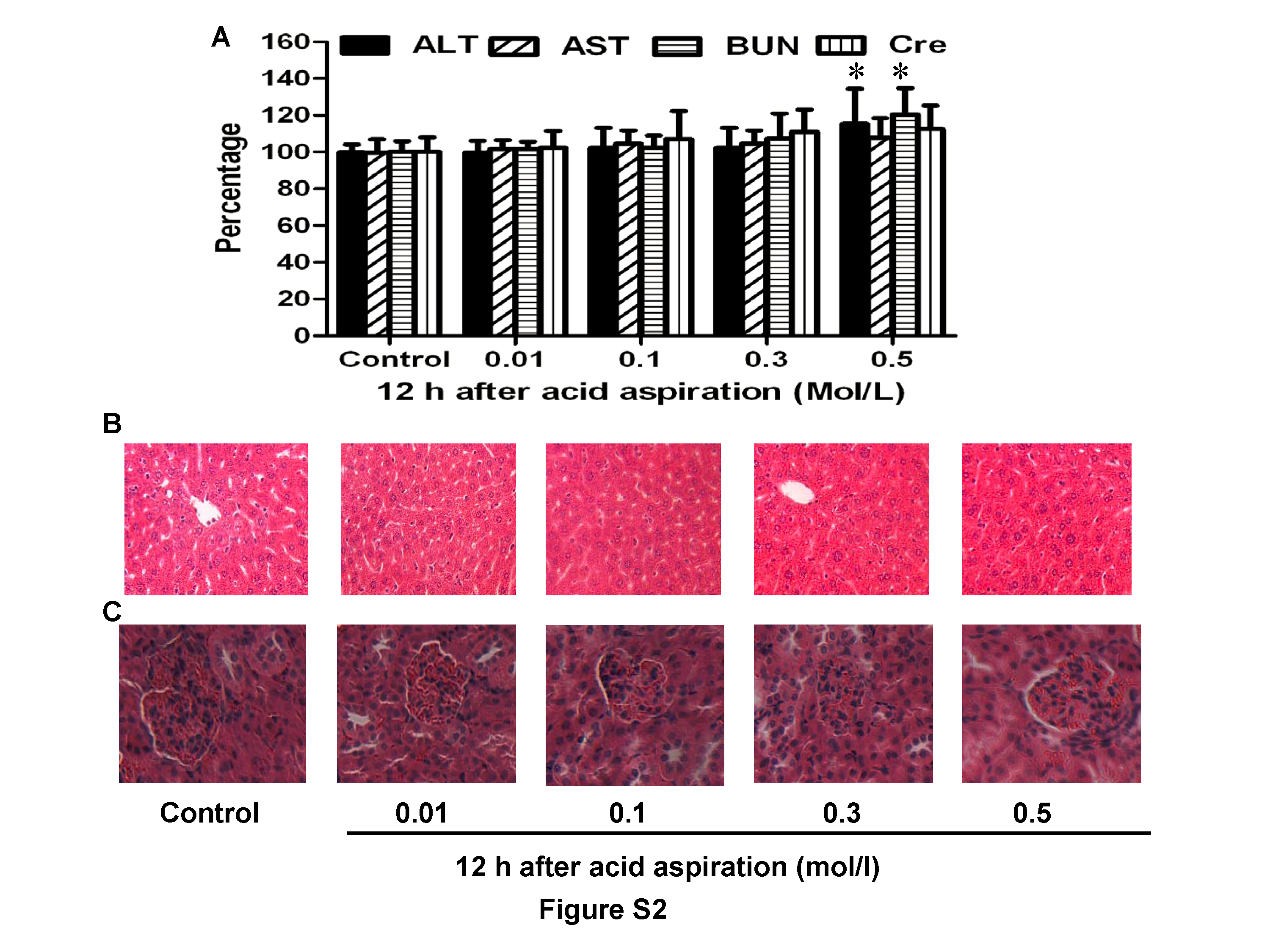

Supplement: Figure S2 — Effect of the concentration of acid aspirated on the function and pathological changes of liver and kidney. After acid aspiration for 12 hours or just before death (n = 7), blood, liver and kidney samples were obtained. Liver (aspartate transaminase, AST; alanine transaminase, ALT) and renal (blood urea nitrogen, BUN; creatinine, Cre) function was analyzed. Mean from control group was designated as 100% and the relative percentages were presented. A slight increase in BUN and AST was seen in the group of 0.5 mol/l (S2A). *p<0.05 vs. the control group. Unlike in the lung, pathological changes in liver and kidney were not obvious, and slight swelling could be seen in liver and kidney tubule cells in the groups of 0.3 and 0.5 mol/l (S2B, S2C). The HE stained sections are representative of 3 similar samples. Original magnification×200. (TIFF) [file pone.0097074.s002.tiff]

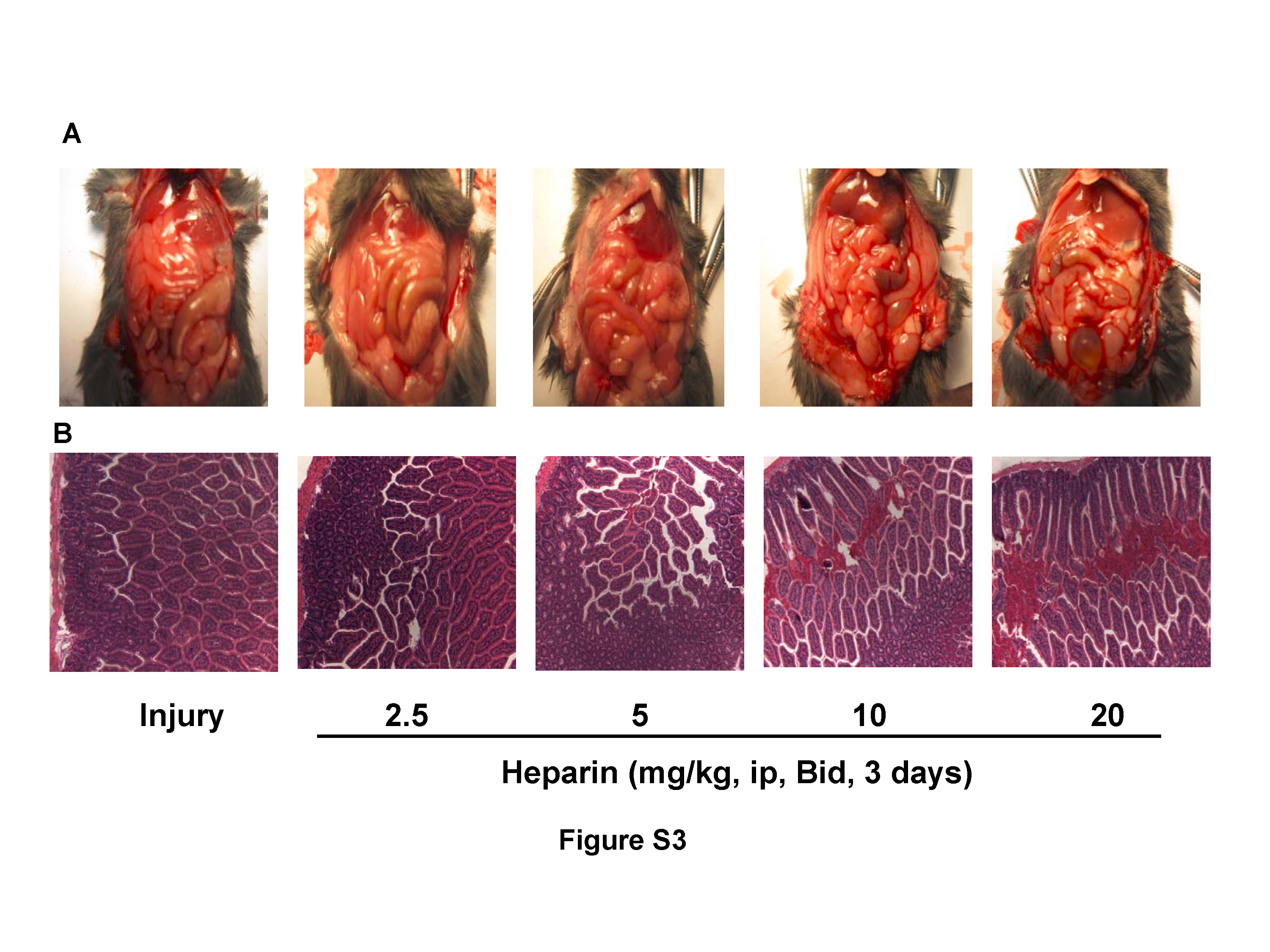

Supplement: Figure S3 — Effect of heparin on abnormal hemorrhage in mice. After intratracheal instillation of HCl (0.3 mol/l, 2 µl g−1), heparin was injected ip, twice a day, for 3 days or until death. The regional necropsies showed that no abnormal hemorrhagic foci were found in the injury group, the 2.5 and 5 mg/kg heparin groups or the NAH groups. Gross view showed that 2 mice (2/14) had disseminated hemorrhage in abdominal cavity in 10 mg/kg heparin group while 5 mice (5/14) had disseminated hemorrhage in the 20 mg/kg heparin group (S3A). Meanwhile ileum samples were obtained for histopathology analysis. The multifocal regions of hemorrhage were present within the muscular intestinal wall in the 10 and 20 mg/kg heparin groups. The HE stained sections are representative of 3 similar samples (S3B). Original magnification×100. (TIFF) [file pone.0097074.s003.tiff]
